# Variable Range Hopping Transport Probed by DNA Sensing in Vertical Graphene and Nanocrystalline Graphite BioFETs

**DOI:** 10.3390/mi17060737

**Published:** 2026-06-18

**Authors:** Marioara Avram, Tiberiu Burinaru, Andrei Avram, Eugen Chiriac, Catalin Marculescu, Bianca Adiaconita

**Affiliations:** National Institute for Research and Development in Microtechnologies—IMT Bucharest, 126A Erou Iancu Nicolae, 077190 Voluntari, Romania; marioara.avram@imt.ro (M.A.); tiberiu.burinaru@imt.ro (T.B.); andrei.avram@imt.ro (A.A.); eugen.chiriac@imt.ro (E.C.); catalin.marculescu@imt.ro (C.M.)

**Keywords:** vertical graphene, nanocrystalline graphite, variable range hopping, BioFET, DNA detection

## Abstract

Biosensing performance in graphene-derived field-effect transistors (BioFETs) is widely attributed to surface chemistry, yet the role of the underlying charge transport mechanism remains poorly understood. This work establishes a direct correlation between disorder-driven transport and biosensing transduction in vertical graphene (VG) and nanocrystalline graphite (NCG) FET devices. Temperature-dependent electrical characterization (15–500 K) reveals a hybrid transport regime: three-dimensional Mott variable-range hopping below 240 K, transitioning to thermally activated Arrhenius-type conduction above 240 K. The extracted VRH parameters characteristic temperature T_0_, localization length ξ, and density of states N(E_F_) quantify fundamentally distinct disorder landscapes: VG operates in a strongly localized, edge-dominated regime, while NCG forms a continuous percolative network with greater transport stability. Surface functionalization via PASE and amine-terminated ssDNA probes, followed by DNA hybridization across four nucleobase systems, demonstrates that the sequence-dependent electrical response is mechanistically interpretable within the VRH–transconductance framework. NCG transduces biomolecular binding through direct charge transfer and hopping pathway perturbation, whereas VG responds through interfacial electrostatic reorganization. These results introduce a unified VRH–transconductance–sensing framework, providing a rational physical basis for next-generation graphene BioFET design.

## 1. Introduction

Graphene and graphene-derived nanomaterials have attracted considerable interest as active channel materials in field-effect transistor biosensors (BioFETs), owing to their high carrier mobility, large accessible surface area, and strong sensitivity to electrostatic perturbations at the solid–liquid interface [1,2]. These properties make them particularly suited for label-free electrical detection of biomolecular interactions, including nucleic acid hybridization, where surface-induced changes in charge density must be transduced into measurable electrical signals [3]. In practical devices, however, the channel material is rarely pristine graphene, but rather a structurally disordered derivative, such as vertical graphene (VG) or nanocrystalline graphite (NCG), whose transport properties deviate significantly from ideal band-like conduction. The presence of grain boundaries, edge defects, and localized electronic states introduces hopping-dominated transport mechanisms that are absent in ideal graphene, yet are highly sensitive to interfacial perturbations. Understanding how these disorder-driven transport mechanisms couple with biomolecular recognition events is therefore essential for the rational design of high-performance graphene-based BioFET platforms [4,5,6,7,8]. Beyond nucleic acid detection, graphene FET platforms have demonstrated remarkable versatility across biomedical sensing contexts, including real-time non-destructive monitoring of organoid drug responses [9] and ultrasensitive viral pathogen detection via recognition of enzymatic activity [10]. Graphene has also attracted significant interest in photonic and electromagnetic wave engineering, including the realization of broadband, wide-angle, and dynamically tunable THz absorbers [11], further underscoring the multifunctional character of graphene-derived materials across device platforms beyond bioelectronics. Recent advances in graphene interface engineering including nano-crumpled graphene architectures for multimodal biosensing [12] have demonstrated that nanostructural design of the graphene surface can substantially enhance biomolecular transduction sensitivity and enable multiplexed readout.

Among these disordered graphene derivatives, VG and NCG have emerged as promising candidates for BioFET applications due to their compatibility with scalable microfabrication techniques and tunable structural properties [13,14]. VG consists of a three-dimensional network of vertically aligned graphene sheets with a high density of exposed edges and large accessible surface area, which enhances biomolecular adsorption and interfacial coupling [15,16,17,18]. In contrast, NCG forms a compact and continuous nanocrystalline graphitic film, providing improved electrical continuity and more stable charge transport pathways [19,20,21]. Despite sharing a common graphitic composition, these two materials exhibit fundamentally different structural organizations, which are expected to produce distinct transport regimes and sensing transduction mechanisms.

From a transport perspective, VG and NCG deviate significantly from ideal band-like conduction due to structural disorder, grain boundaries, and localized electronic states. In such systems, charge transport is governed by hopping-based mechanisms, particularly three-dimensional Mott variable range hopping (3D VRH) at low temperatures, in which carriers tunnel between localized states with the assistance of phonons [22,23,24,25,26,27]. Table 1 summarizes VRH transport parameters reported in the literature for representative disordered carbon-based systems, confirming that 3D Mott VRH is the dominant low-temperature conduction mechanism across graphene-derived and carbon nanotube materials, with transport dimensionality governed by structural morphology. Although hybrid VRH–thermally activated regimes have been previously reported in analogous graphitic systems, a systematic framework correlating these transport parameters with biosensing performance in VG- and NCG-based BioFETs remains absent from the literature, a gap addressed by the present work.

The characteristic temperature (T_0_), localization length (ξ), and density of states at the Fermi level (N(E_F_)) extracted from VRH analysis provide quantitative descriptors of the disorder landscape, enabling direct comparison between materials with different structural organization. The role of structural dimensionality in determining the dominant VRH regime has been demonstrated in reduced graphene oxide systems, where two-dimensional assemblies follow Mott’s 2D VRH model, while three-dimensional nanostructures transition to 3D VRH, confirming that artificial 3D architectures at the nanoscale directly modify electron conduction behavior [28]. Similar behavior has been reported in vertically aligned carbon nanotube films, where a combination of inter-tube hopping and graphitic conduction governs resistivity, with the VRH dimensionality depending on film thickness and network connectivity [29]. In graphitic carbon materials derived from polyaniline, 3D VRH dominates at low temperatures, transitioning to inter-domain hopping at elevated temperatures [30], a hybrid transport regime directly analogous to that observed in VG and NCG channels in the present work.

Despite these precedents in carbon-based systems, a unified theoretical and experimental framework describing the electrical behavior of newer 3D graphene-derived nanomaterials, specifically VG and NCG, remains largely absent from the literature. Critically, these hopping-dominated transport pathways are inherently sensitive to nanoscale perturbations at the graphene interface, suggesting that VRH modulation may serve as a primary transduction mechanism in graphene-based BioFETs, a possibility that has received little systematic attention.

The present study addresses this gap by establishing a direct correlation between VRH transport parameters and DNA hybridization transduction in VG- and NCG-based BioFETs.

The present study constitutes a mechanistic transport-biointerface correlation study; analytical performance characterization—including limit of detection, dynamic range, and selectivity against non-complementary sequences—is outside the scope of this work and represents a natural direction for subsequent studies.

## 2. Materials and Methods

### 2.1. Reagent and Device Fabrication

All reagents were purchased from Sigma-Aldrich Co., LLC, (St. Louis, MO, USA). with the exception of oligonucleotides, which were supplied by Integrated DNA Technologies. The oligonucleotide probes used in this study are summarized in Table 2. All probes were single-stranded DNA homopolymers of 20 bases: PolyG, PolyC, PolyA, and PolyT, each composed exclusively of a single nucleotide type and functionalized with a 5′ Amino Modifier C6 group to enable covalent coupling with the NHS ester moiety of PASE through stable amide bond formation. Probes were supplied at a concentration of 100 µM in IDTE buffer (pH 8.0). The melting temperatures of the G–C probes (PolyG and PolyC, Tm = 78.7 °C) and A–T probes (PolyA and PolyT, Tm = 37.3 °C) reflect their differential duplex stability and directly correlate with the sequence-dependent electrical responses observed in the transfer characteristics of both VG- and NCG-based FET devices.

The devices were fabricated as FETs, in which the conductive channel between the source and drain electrodes consisted of graphene-derived nanomaterials. Two types of channel materials were investigated: VG and NCG, selected due to their distinct structural organization and electronic transport properties.

The fabrication procedures, structural characteristics, and baseline electrical performance of the NCG-based FET devices have been previously reported in detail [31,32]. In the present work, VG-based FET devices were fabricated following the same technological approach and microfabrication protocol, ensuring comparable device architecture and electrode configuration. Complete microfabrication details, device schematics, and baseline electrical characterization are provided in Refs. [31,32], to which the reader is referred for full methodological context. A direct comparative electrical characterization of graphene materials- based FETs fabricated on identical substrates is available in Ref. [31], which establishes the baseline electrical differences between these materials; a VRH analysis of SLG is not applicable in the present framework, as band-like conduction dominates in the ordered sp^2^ lattice of single-layer graphene.

The graphene-based channel was integrated between metallic source and drain electrodes, forming a planar FET architecture. The application of a gate voltage modulates the carrier concentration within the channel, enabling the extraction of transfer characteristics (I_D_–V_G_) and the determination of the Dirac point. Temperature-dependent current–voltage (I–V) characteristics were recorded over the range 15–500 K for both NCG- and VG-based FET structures, enabling the identification of dominant charge transport mechanisms and the extraction of key transport parameters.

Due to their distinct morphologies and electronic structures, VG and NCG are expected to exhibit different transduction mechanisms. NCG, characterized by a compact, bulk-like three-dimensional structure in which graphene sheets are densely interconnected, favors charge-transfer-dominated interactions and relatively delocalized carrier transport. In contrast, VG with its vertically oriented nanowall architecture and high density of exposed edge sites promotes enhanced interfacial coupling, increased capacitive contributions, and heightened sensitivity to surface-induced electrostatic perturbations.

### 2.2. Surface Functionalization and DNA Hybridization

Surface functionalization of the VG and NCG channels was performed via non-covalent anchoring using 1-pyrenebutyric acid N-hydroxysuccinimide ester (PASE), through π–π interactions between the pyrene aromatic core and the carbon material surface. Prior to functionalization, FET devices were cleaned with isopropanol and dried at room temperature for 10 min under laminar flow conditions in an ISO 14644-1 Class 6 cleanroom, (IMT Bucharest, Bucharest, Romania).

PASE solution (5 µL, 10 mM in DMF) was drop-cast onto the VG/NCG channel surface and incubated at room temperature for 24 h to ensure effective adsorption. Following functionalization, devices were rinsed sequentially with DMF and deionized water to remove unbound molecules, then dried at room temperature.

Immobilization of single-stranded DNA probes (PolyG, PolyC, PolyA, and PolyT, 3′-NH_2_) was achieved through covalent coupling of the terminal amine groups with the NHS ester functionality of PASE. A volume of 5 µL of ssDNA solution (100 µM in IDTE buffer, 1 × TE, pH 8) was applied to the functionalized surface and incubated at room temperature for 24 h. After incubation, devices were rinsed three times with IDTE buffer to remove unreacted probes, followed by three washes with deionized water, and dried at room temperature. The ssDNA probe concentration of 100 µM was selected consistently with previously reported functionalization protocols for VG-based sensing interfaces, in which this concentration was shown to ensure maximum surface coverage on the three-dimensional VG architecture [14].

Hybridization was performed by exposing the functionalized devices to complementary ssDNA (PolyC for PolyG probes; PolyT for PolyA probes) at a concentration of 100 µM for 4 h at room temperature. Devices were subsequently rinsed three times with IDTE buffer to remove non-hybridized strands and dried prior to electrical characterization. Among the nucleobases investigated, guanine exhibited the strongest interaction with the VG surface, consistent with its highest HOMO energy level relative to the Fermi level of the carbon channel, which favors preferential charge transfer and π–π electronic coupling.

### 2.3. Electrical Characterization and Temperature-Dependent Measurements

Electrical characterization of the FET devices was performed using a semiconductor parameter analyzer (4200-SCS, Keithley Instruments, Cleveland, OH, USA). The transfer characteristics (I_D_–V_G_) were recorded at a constant drain voltage (V_D_ = 2 V), enabling the evaluation of carrier transport behavior and the identification of the Dirac point.

To investigate the underlying charge transport mechanisms, temperature-dependent measurements were carried out over a wide temperature range. This approach allows the identification of dominant conduction regimes, including thermally activated transport and variable range hopping, as well as the assessment of disorder-related effects such as carrier localization and tunneling processes in VG and NCG channels.

The sensing performance of the devices was evaluated by monitoring the evolution of the Dirac point after each functionalization step, namely: (i) bare graphene channel, (ii) PASE-modified surface, (iii) ssDNA immobilization, and (iv) dsDNA formation following hybridization. The shifts in the Dirac point reflect changes in the local electrostatic environment and charge distribution at the graphene interface, and provide a direct electrical readout of biomolecular interactions.

### 2.4. Structural and Morphological Characterization

The morphological characterization of the graphene-based channels (VG and NCG) was performed using field-emission scanning electron microscopy (FE-SEM, NOVA NanoSEM 630, Thermo Fisher Scientific, Hillsboro, OR, USA), enabling the evaluation of surface topology, structural uniformity, and the distribution of graphene features.

Structural analysis of the carbon materials, as well as the assessment of lattice integrity following surface functionalization and biomolecular interactions, were carried out by Raman spectroscopy. Particular attention was given to the evolution of the characteristic D (~1350 cm^−1^), G (~1580 cm^−1^), and 2D (~2700 cm^−1^) bands, which provide insight into defect density, graphitic order, and electronic structure.

Changes in the relative intensity and position of these bands were used to evaluate the degree of disorder, functionalization-induced perturbations, and possible charge transfer effects at the graphene–bio interface.

## 3. Results

Graphene-based BioFET sensors have attracted considerable attention for label-free nucleic acid detection, owing to their high carrier mobility and strong sensitivity to surface charge perturbations [18,19,20]. In the conventional interpretation, DNA sensing is described as an electrostatic gating effect: the negatively charged phosphate backbone of immobilized DNA strands modulates the carrier density in the graphene channel and induces a measurable shift of the Dirac point. While this framework is adequate for pristine graphene, it is insufficient to account for the electrical response observed in structurally disordered materials such as VG and NCG, where charge transport is governed by hopping between localized states rather than delocalized band conduction. In such systems, the transition from bare graphene to a functionalized biointerface, through sequential PASE adsorption, single-stranded DNA (ssDNA) immobilization, and double-stranded DNA (dsDNA) hybridization, must be understood not only as an electrostatic perturbation, but as a progressive modification of the disorder landscape governing VRH conduction. Each functionalization step alters the tunneling distance, the local density of localized states, and the interfacial potential profile, directly affecting the hopping pathways and thermally activated transport barriers. The transition from ssDNA to dsDNA is particularly significant: the structural reorganization from a flexible single-stranded layer to a rigid double helix modifies the charge distribution at the graphene interface in a manner that depends on the specific nucleobase composition. The distinct stacking energies and dipole moments of G–C versus A–T base pairs result in differential electronic coupling to the graphene surface, producing base-specific modulation of transport parameters.

This coupling between biomolecular recognition and disorder-driven transport is expected to manifest differently in VG and NCG. In NCG, the more continuous and ordered conductive network favors charge-transfer-dominated interactions, producing stable and reproducible modulation of channel conductance upon DNA binding, predominantly reflected in I_ON_/I_OFF_ variations rather than significant Dirac point displacement. In VG, the edge-rich, three-dimensional architecture and strongly localized transport regime reflected in its shorter ξ (ξ = 1.5 nm, vs. 3.0 nm for NCG) amplifies sensitivity to interfacial perturbations, resulting in pronounced current modulation that reflects modifications of hopping pathways rather than simple electrostatic gating. A rigorous physical interpretation of DNA detection in these materials therefore requires a transport modeling framework combining VRH analysis and thermally activated conduction that can resolve these distinct transduction mechanisms and connect them quantitatively to the biomolecular events at the graphene interface.

### 3.1. Charge Transport Modeling in NCG/VG

In semiconductor materials, electrical conductivity increases with temperature, in contrast to metals where conductivity decreases due to enhanced electron–phonon scattering. For both graphene-based materials investigated VG and NCG the conductivity exhibits a clear positive temperature dependence over the entire investigated range (15–500 K), consistent with thermally activated transport and hopping conduction mechanisms.

Electrical characterization was performed by measuring current–voltage (I_D_–V_G_) transfer characteristics over a temperature range of 15–500 K for FET structures incorporating NCG and VG as active channel materials. For both types of structures, the I–V characteristics exhibited linear behavior, confirming ohmic contacts between the Au electrodes and the graphene-derived channels [22,23].

The conduction mechanism was analyzed through the following steps: (i) evaluation of the temperature dependence of resistance R(T) or conductivity σ(T) in the low-temperature regime (T < 120 K and T < 240 K); (ii) representation of ln[σ(T)] as a function of T^−1/4^ (3D VRH), T^−1/3^ (2D VRH), and T^−1/2^ (Coulomb gap model); (iii) assessment of linearity to identify the dominant transport mechanism; (iv) extraction of the characteristic temperature T_0_ from the slope of the linear fits, followed by estimation of the localization length (ξ) and the density of states at the Fermi level, N(E_F_).

A high T_0_ value indicates a high degree of structural disorder and reduced charge transport efficiency. Correspondingly, a small ξ reflects strong carrier localization, while a low density of states at the N(E_F_) indicates weak doping and/or the presence of an effective energy gap.

### 3.2. Temperature Dependence of Resistivity and Conductivity

**Figure 1:**
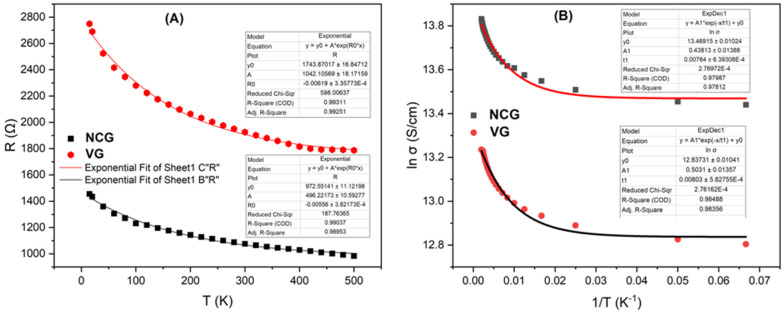
Variation in electrical resistance as a function of temperature (**A**) and variation in the natural logarithm of conductivity as a function of inverse temperature (**B**) for FET structures based on NCG (black) and VG (red).

Figure 1 presents the temperature dependence of electrical resistance for NCG- and VG-based FET structures over the full temperature range (15–500 K). Both materials show a thermally activated decrease in resistance over the temperature interval, consistent with hopping-dominated transport. VG exhibits a resistance decrease from ~2.8 to ~1.8 kΩ, while NCG decreases from ~1.4 to ~0.9 kΩ over the same range. The reduction in resistance with increasing temperature observed in both VG and NCG does not reflect conventional band-like semiconductor transport, but instead indicates thermally activated hopping conduction within structurally disordered carbon networks. Although the two materials exhibit distinct morphologies and structural ordering, charge transport in both systems is governed by localized electronic states associated with defects, disorder, and grain boundaries. As a result, their electrical behavior follows a variable range hopping (VRH) mechanism, characteristic of disordered carbon-based transport regimes.

A significant change in the slope of the resistance–temperature dependence is observed depending on the temperature region, indicating distinct conduction mechanisms at low temperatures (T < 240 K) and high temperatures (T > 240 K). This transition suggests a shift from hopping-dominated transport in the low-temperature regime to thermally activated conduction at elevated temperatures.

Across the entire investigated temperature range, the source–drain resistance of VG-based devices is consistently higher than that of NCG, reflecting increased structural disorder and reduced carrier transport efficiency in vertically oriented graphene. At temperatures above room temperature, the resistance values of the two materials converge, indicating that thermal activation becomes the dominant mechanism in both cases.

**Figure 2:**
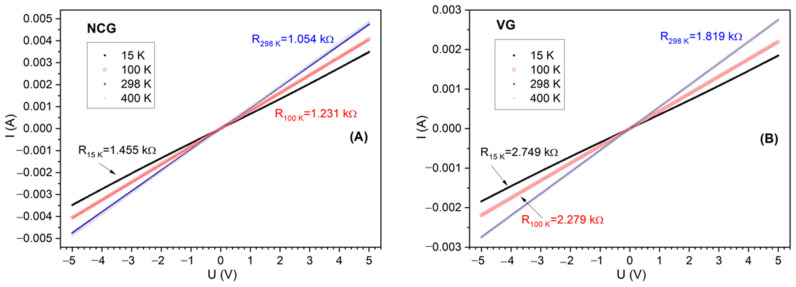
I–V characteristics for NCG-FET (**A**) and VG-FET (**B**) at representative temperatures: 15 K, 100 K, 298 K, 400 K.

In Figure 2, we present the I–V characteristics of NCG-FET and VG-FET obtained at specific temperatures.

**Figure 3:**
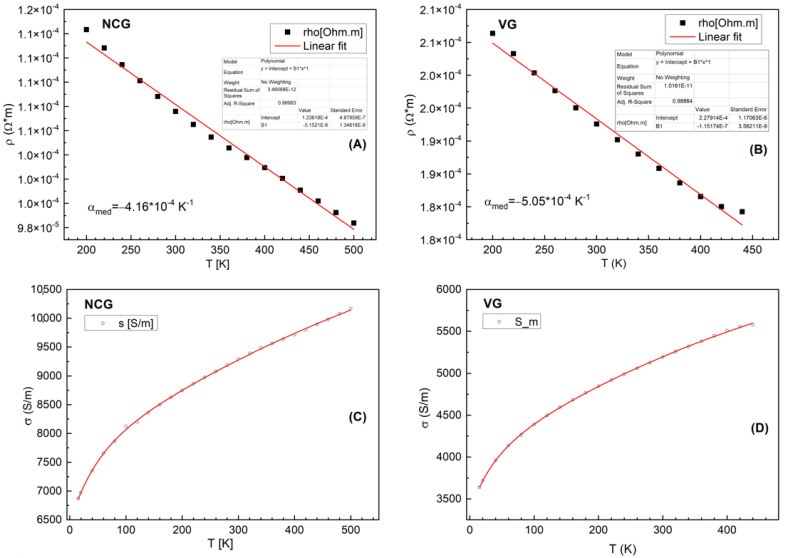
Temperature dependence of resistivity and electrical conductivity for FET structures based on NCG and VG: (**A**) temperature dependence of resistivity for NCG; (**B**) temperature dependence of resistivity for VG; (**C**) temperature dependence of electrical conductivity for NCG; (**D**) temperature dependence of electrical conductivity for VG.

Figure 3 illustrates the temperature dependence of electrical resistivity and electrical conductivity in the high-temperature region (T > 200 K). For temperatures above 200 K, resistivity exhibits an approximately linear dependence on temperature. The extracted temperature coefficients are α = −4.16 × 10^−4^ K^−1^ for NCG and α = −5.05 × 10^−4^ K^−1^ for VG. The temperature coefficients are negative, and in absolute value they are an order of magnitude smaller than in metals, such as Cu, Au, or Ag. The electrical conductivity of NCG is almost double that of VG over the entire temperature range.

### 3.3. Transport Modeling: 3D VRH and Thermally Activated Conduction

**Figure 4:**
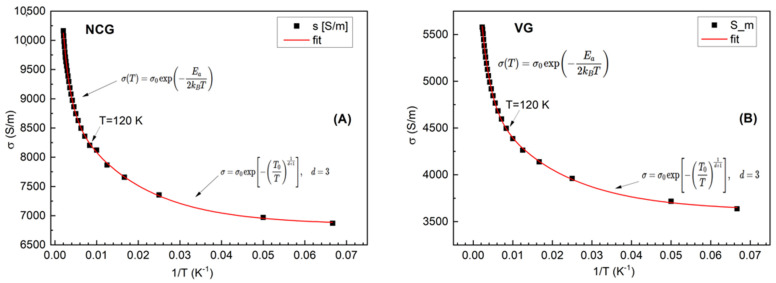
Arrhenius and VRH models used to analyze the temperature-dependent conductivity of (**A**) NCG-based FET and (**B**) VG-based FET.

Figure 4 presents the analysis of the temperature dependence of conductivity using two complementary models:

(1) The variable range hopping (VRH) model, relevant at low temperatures (typically T < 240 K), described by:

(1) $$\sigma =\sigma_{0}{exp[-\left(T_{0}/T\right)}^{\frac{1}{d+1}}]$$

(2) The thermally activated transport model, dominant at higher temperatures (T > 240 K), expressed as:

(2) $$\sigma =\sigma_{0}exp(-E_{a}/2k_{B}T)$$

Here, T_0_ represents the characteristic temperature, *d* is the effective dimensionality of the system, and *E*_a_ denotes the activation energy. For both NCG and VG structures, the best fit was obtained for *d* = 3, indicating a three-dimensional hopping transport mechanism, consistent with the structural morphology of both materials [28,29].

### 3.4. Low-Temperature VRH Analysis: Extracted Parameters and Physical Interpretation

As established in Section 3.2, VG and NCG operate in a hybrid transport regime—3D Mott VRH below 240 K transitioning to thermally activated conduction above 240 K—with fundamentally distinct disorder landscapes (VG: ξ = 1.5 nm, high N(E_F_), short R_hop_; NCG: ξ = 3.0 nm, stable T_0_, lower N(E_F_)) that directly govern their respective biosensing transduction mechanisms.

**Figure 5:**
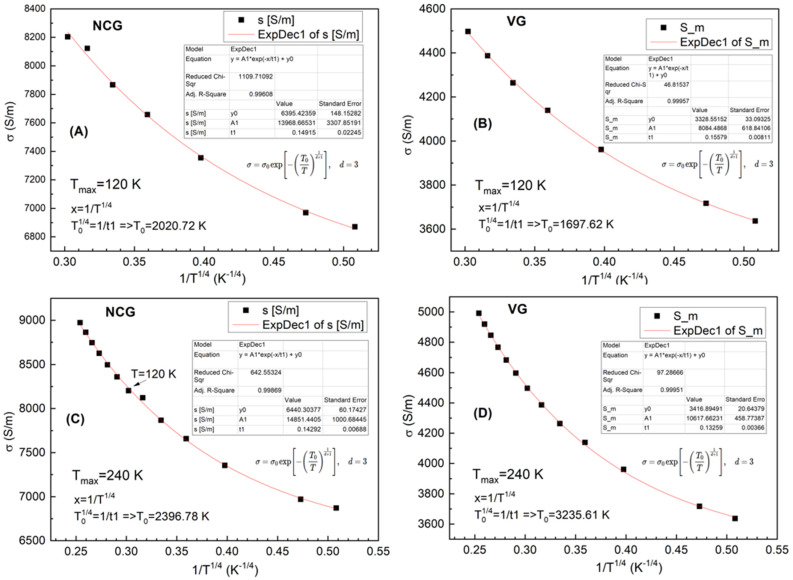
VRH analysis of conductivity in the low-temperature regime for NCG and VG FET devices, plotted as σ versus T^−1/4^ (d = 3), confirming disorder-driven transport and carrier localization effects: (**A**) VRH analysis for NCG at T_max_ = 120 K; (**B**) VRH analysis for VG at T_max_ = 120 K; (**C**) VRH analysis for NCG at T_max_ = 240 K; (**D**) VRH analysis for VG at T_max_ = 240 K.

The best linearity was obtained for the ln(σ) versus T^−1/4^ representation (Figure 5), confirming that charge transport in both NCG and VG is well described by the 3D Mott VRH mechanism.

Fitting of the low-temperature conductivity data (T < 120 K) using the 3D Mott VRH model (Equation (1)) with a function of the form exp [−(T_0_/T)^1/4^] yields the following characteristic temperatures: T_0_ = 2020.72 K for NCG and T_0_ = 1697.62 K for VG in the pure VRH regime. These values are in agreement, in terms of order of magnitude, with characteristic temperatures reported for related disordered carbon-based systems [28].

From the characteristic temperature, the density of states at the Fermi level *N*(*E*_F_) can be estimated using the relation:

(3) $${\;T}_{0}=\;\frac{\beta }{k_{B}\cdot N\left(E_{F}\right){\cdot \xi }^{d}}$$

where β ≈ 21 is a numerical prefactor for the 3D Mott model, *k_B_* is the Boltzmann constant, and ξ is the carrier localization length. Using the experimentally determined values ξ = 3.0 nm for NCG and ξ = 1.5 nm for VG, the extracted parameters are summarized in Table 3.

The extracted VRH transport parameters summarized in Table 3 reveal a fundamental physical distinction between VG and NCG that directly governs their biosensing behavior. For VG (ξ = 1.5 nm), the characteristic temperature T_0_ increases sharply from 1697 K (T_max_ = 120 K) to 3235 K (T_max_ = 240 K), a 90.6% increase, indicating that the pure Mott VRH regime breaks down above 120 K as competing transport mechanisms become active, likely associated with the strongly fragmented, edge-dominated conduction network of VG. In contrast, NCG (ξ = 3.0 nm) exhibits a stable T_0_ transition from 2021 K to 2397 K (+18.6%), confirming that a single VRH mechanism governs transport over the entire low-temperature range, consistent with its more continuous percolative network.

The density of states at the Fermi level is estimated as N(E_F_) ≈ 4.5 × 10^21^ eV^−1^cm^−3^ for NCG and N(E_F_) ≈ 4.3 × 10^22^ eV^−1^cm^−3^ for VG at T_max_ = 120 K. Although VG exhibits a higher T_0_ in the extended fit, its N(E_F_) is approximately one order of magnitude larger than that of NCG, an apparently paradoxical result that is physically consistent. The structural disorder in VG, manifested through a high density of edge sites, grain boundaries, and inter-sheet junctions, generates a correspondingly large density of localized states within the pseudogap, which are precisely the states that mediate hopping conduction and, subsequently, biomolecular transduction. The mean hopping distance R_hop_ = (3/8)·ξ·(T_0_/T)^1/4^ further quantifies this distinction: VG exhibits R_hop_ ≈ 2.91 nm, while NCG exhibits R_hop_ ≈ 6.08 nm at T_max_ = 120 K. The shorter hopping distance in VG means that each carrier hop is spatially confined to a region comparable to the dimensions of a single adsorbed biomolecule, rendering the transport network inherently sensitive to localized interfacial perturbations. In NCG, the longer R_hop_ implies that individual hopping events sample a larger spatial region, averaging out local perturbations and resulting in a more stable but less sensitive electrical response.

Taken together, the combination of short ξ, high N(E_F_), and short R_hop_ in VG establishes a transport regime in which biomolecular adsorption of PASE, ssDNA, or dsDNA directly and efficiently modulates the dominant conduction pathways, amplifying the biosensing response. In NCG, the larger ξ and R_hop_ combined with lower N(E_F_) shift the transduction mechanism toward dielectric modulation, producing attenuated but reproducible electrical signals. These intrinsic differences in transport parameters provide the physical foundation for the sequence-dependent biosensing behavior observed in the transfer characteristics of both VG- and NCG-based FET devices.

Experimentally, NCG exhibits significantly higher conductivity than VG across the entire temperature range, with values approximately twice as large (σ_NCG_∼10^4^ S/m vs. σ_VG_∼5 × 10^3^ S/m at room temperature). This difference is attributed to their distinct morphologies: NCG consists of interconnected nanometric graphitic domains forming a relatively compact percolative network, while VG is composed of vertically oriented graphene sheets with a fragmented conduction pathway dominated by edges, inter-sheet junctions, and defect-rich regions.

The VRH parameters T_0_, ξ, and N(E_F_) are material-specific structural descriptors that quantify the disorder landscape independently of measurement temperature. Although pure Mott VRH conduction is dominant below 240 K, the localized state distribution characterized by these parameters persists at room temperature and governs the sensitivity of thermally activated transport to interfacial perturbations. At room temperature, both VG and NCG operate in the Arrhenius regime (E_a_ ≈ 0.062–0.065 eV); however, the magnitude of V_Dirac_ shifts and I_ON_/I_OFF_ modulation upon functionalization is determined by the same localized state landscape extracted from the low-temperature VRH analysis. In VG (ξ = 1.5 nm), charge pathways remain spatially confined to regions comparable to individual adsorbed biomolecules (~2–5 nm) at all temperatures, rendering the transport network intrinsically sensitive to surface perturbations regardless of the dominant conduction regime. In NCG (ξ = 3.0 nm), the longer localization length causes charge pathways to sample a larger spatial volume, averaging out local perturbations and producing a more stable but less sensitive response. The VRH parameters therefore serve as rational structural predictors of room-temperature biosensing sensitivity not because VRH conduction is active during sensing, but because they quantify the disorder landscape that governs interfacial sensitivity at any temperature.

It is worth noting that recent nanoengineered graphene platforms, such as the nano-crumpled graphene multimodal biosensing architecture [12], achieve superior analytical sensitivity through geometric surface area amplification and multimodal transduction. The present work addresses a complementary and previously unexplored dimension: the intrinsic disorder-driven transport mechanism quantified through VRH analysis as a physical determinant of transduction specificity. Engineering the localization length, disorder density, and hopping pathway connectivity represents an orthogonal design axis that can, in principle, be combined with surface amplification strategies to yield next-generation graphene BioFET platforms with both high sensitivity and physically interpretable transduction mechanisms.

### 3.5. High-Temperature Transport: Thermally Activated Conduction

**Figure 6:**
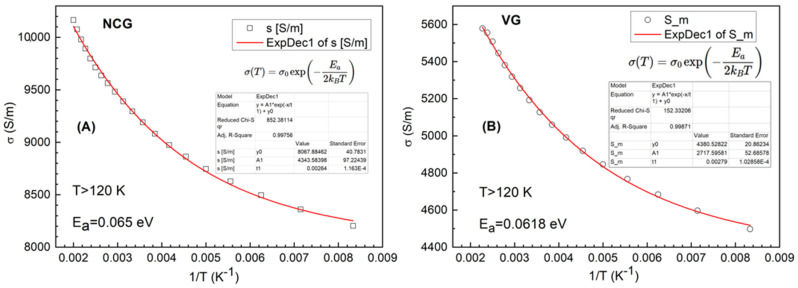
Arrhenius analysis of conductivity for NCG and VG FET devices at T > 120 K, showing thermally activated transport and extracted activation energies: (**A**) Arrhenius analysis for NCG, with E_a_ = 0.065 eV; (**B**) Arrhenius analysis for VG, with E_a_ = 0.0618 eV.

Figure 6 presents the analysis at temperatures above 120 K, when the conductivity behavior follows an Arrhenius-type dependence, which confirms a thermally activated transport. The extracted activation energies are E_a_ = 0.065 eV for NCG and E_a_ = 0.0618 eV for VG, indicating conduction across shallow energy barriers associated with disorder, grain boundaries, and interfacial regions. These values are consistent with pseudogap behavior characteristic of disordered graphene-derived systems, and confirm that both materials behave as semiconductors rather than semimetals in this temperature regime [31,32].

The comparable activation energies suggest a similar high-temperature transport mechanism in both materials. However, the slightly stronger temperature dependence observed in VG (α = −5.05 × 10^−4^ K^−1^ vs. −4.16 × 10^−4^ K^−1^ for NCG) reflects its higher degree of structural disorder and increased contribution of interfacial effects.

Combined with the VRH behavior identified at lower temperatures, these results demonstrate that NCG/VG-FET channels operate in a hybrid transport regime, transitioning from 3D Mott VRH below 240 K to thermally activated conduction above 240 K. This behavior is consistent with disorder-driven transport models in which conduction is controlled by the interplay between energy barriers, tunneling processes, and network connectivity.

### 3.6. Unified Transport Framework and Implications for BioFET Sensing

The combined temperature-dependent analysis supports a unified transport mechanism governed by a VRH–tunneling framework. At low temperatures (T < 240 K), charge transport is dominated by 3D Mott VRH between localized states, while at higher temperatures (T > 240 K), thermally activated transport and tunneling across potential barriers become increasingly relevant.

A clear distinction between VG and NCG emerges from the extracted parameters. VG exhibits higher T_0_ (in the extended regime), shorter localization length (ξ = 1.5 nm vs. 3.0 nm), shorter mean hopping distance (R_hop_ = 2.91 nm vs. 6.08 nm), and a one order of magnitude higher N(E_F_), indicating stronger carrier localization and a disorder-dominated transport regime. NCG, in contrast, shows greater T_0_ stability, longer localization length, and lower N(E_F_), consistent with a more continuous percolative network.

These findings have direct implications for BioFET sensing. The introduction of a biofunctional layer (PASE, ssDNA, dsDNA) modifies the tunneling distance, local electrostatic potential, and density of localized states all parameters that directly govern VRH conduction. DNA hybridization, in particular, induces structural and charge redistribution at the graphene interface, affecting both hopping pathways and tunneling barriers. Due to its disorder-dominated transport regime and higher N(E_F_), VG is inherently more sensitive to such interfacial perturbations, which leads to amplified electrical responses upon biomolecular interactions. In contrast, NCG provides improved stability and reproducibility due to its more continuous conductive network.

#### 3.6.1. Electrical Response of VG upon DNA Functionalization

**Figure 7:**
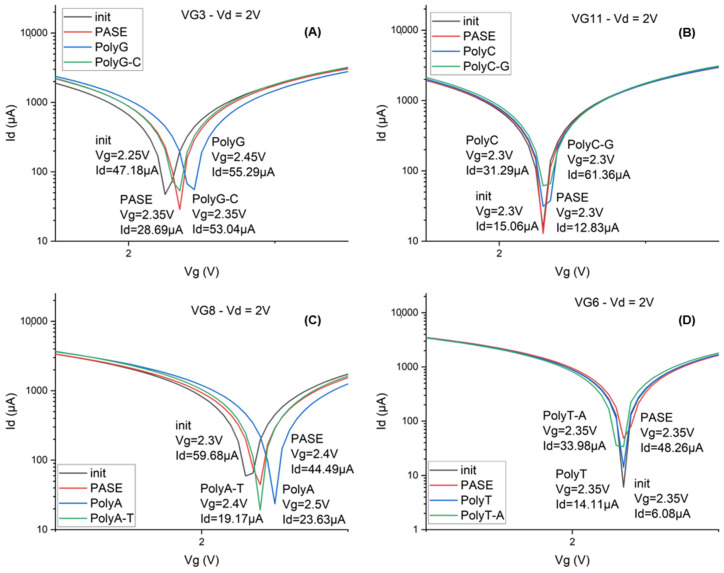
Transfer characteristics of VG-FET (V_D_ = 2 V) showing Dirac point shifts and current modulation upon PASE functionalization, ssDNA immobilization, and DNA hybridization: (**A**) PolyG; (**B**) PolyC; (**C**) PolyA; (**D**) PolyT.

The transfer characteristics (I_D_–V_G_) of VG-FET devices (Figure 7) exhibit a typical ambipolar behavior, with a well-defined Dirac point that shifts progressively following each functionalization step. The sequential modification of the channel—PASE functionalization, ssDNA immobilization, and DNA hybridization—induces systematic changes in both the position of the Dirac point and the minimum channel current, confirming the sensitivity of VG to interfacial perturbations.

PASE functionalization produces a consistent shift of the Dirac point accompanied by a reduction in the minimum current, indicating p-type doping induced by π–π interactions between the pyrene moiety and the graphene surface. The subsequent immobilization of ssDNA introduces additional electrostatic gating effects due to negatively charged phosphate groups, leading to further modulation of the carrier density and a shift in the charge neutrality point.

DNA hybridization induces a distinct electrical response, characterized either by stabilization or additional displacement of the Dirac point, depending on the nucleotide sequence, along with changes in the minimum current. These variations reflect the transition from a flexible ssDNA layer to a more rigid dsDNA structure, which alters both the local dielectric environment and the charge distribution at the interface.

Sequence-dependent behavior is clearly observed. Devices functionalized with PolyG–PolyC exhibit the strongest modulation, consistent with enhanced π–π interactions and higher duplex stability, resulting in pronounced Dirac point shifts and current variation. In contrast, PolyA–PolyT systems show weaker responses, reflecting lower binding strength and reduced electronic coupling. In some cases, particularly for PolyC-based systems, the sensing mechanism is dominated by current modulation rather than significant energetic shifts, indicating a stronger capacitive contribution.

The extracted I_ON_/I_OFF_ ratio reveal distinct electrical signatures for each nucleobase system (Table 4). Notably, G–C and C–G systems exhibit a decrease in I_ON_/I_OFF_ ratio upon hybridization, whereas A–T and T–A systems maintain or increase the ratio, indicating base-specific modulation of the graphene channel conductivity. This behavior supports the hypothesis of localized pseudogap (E_a_) formation and density-of-states N(E_F_) modulation induced by biomolecular interactions.

The electrical parameters extracted at V_D_ = 2V reveal a pronounced sequence-dependent modulation of both V_Dirac_ and I_ON_/I_OFF_, consistent with the disorder-dominated VRH transport regime of VG (ξ ≈ 1.5 nm, high N(E_F_)).

PASE functionalization produces p-type doping in VG3 and VG8 (ΔV_Dirac_ = +0.10 V), reflected in a positive shift of the charge neutrality point and a reduction of I_D_,min, while VG11 and VG6 respond exclusively through current modulation. This device-dependent variability is attributed to the preferential adsorption of the pyrene moiety at edge sites and inter-sheet junctions, the dominant hopping pathways in VG whose local occupation selectively perturbs the most conductive channels of the VRH network.

ssDNA immobilization amplifies the Dirac point displacement in PolyG- and PolyA-based devices (ΔV_Dirac_ = +0.20 V), driven by electrostatic gating from the negatively charged phosphate backbone acting directly on localized carrier states. PolyC and PolyT probes produce no measurable V_Dirac_ shift, indicating a more uniform charge distribution that modifies the effective dielectric environment of hopping sites rather than their energy a distinction that is directly resolvable within the VRH framework through the differential response of V_Dirac_ versus I_D_,min.

DNA hybridization produces the most diagnostically significant and sequence-resolved response. G–C systems (VG3) engage both electrostatic and transport modulation mechanisms: the high-stability G–C duplex perturbs localized state energies and tunneling distances simultaneously, producing correlated changes in V_Dirac_ and I_ON_/I_OFF_. C–G and T–A systems (VG11, VG6) operate through capacitive dielectric screening, with large I_ON_/I_OFF_ modulation (172.6 → 48.9 and 263.2 → 53.0, respectively) in the absence of any V_Dirac_ shift, a signature of duplex-induced modification of the tunneling barrier height without net charge transfer. A–T systems (VG8) represent an intermediate regime, with small but reproducible Dirac point recovery (ΔV = −0.10 V upon hybridization) and near-constant I_ON_/_IOFF_ (73.0 → 71.9), reflecting the weaker electronic coupling of the A–T duplex with the VG hopping network.

Collectively, these results establish that VG-based FET devices provide a multi-parameter electrical fingerprint for DNA hybridization, in which V_Dirac_ encodes the net charge transfer at the interface and I_ON_/I_OFF_ encodes the dielectric modulation of the conductive network two mechanisms whose relative contribution is sequence-specific and directly interpretable through the transport parameters ξ, N(E_F_), and T_0_ extracted from the VRH analysis.

#### 3.6.2. Electrical Response of NCG-FETs upon DNA Functionalization

**Figure 8:**
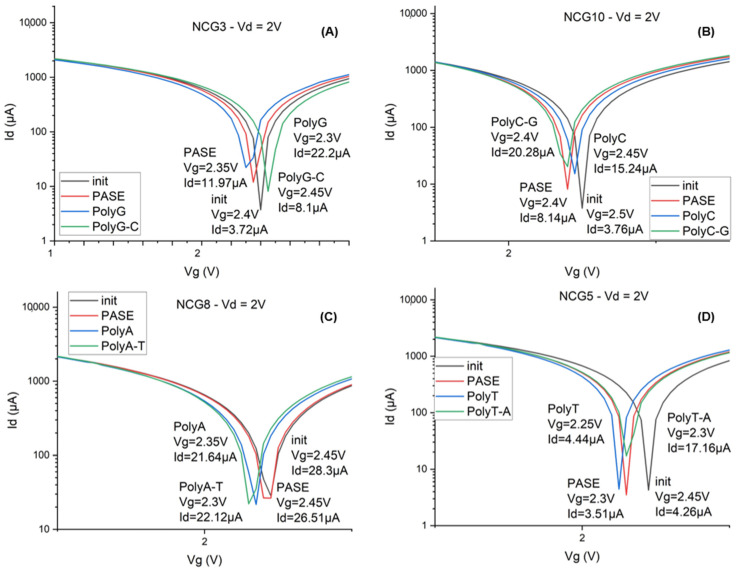
Transfer characteristics of NCG-FETs (V_D_ = 2 V) showing reduced Dirac point shifts and current modulation upon PASE functionalization, ssDNA immobilization, and DNA hybridization: (**A**) PolyG; (**B**) PolyC; (**C**) PolyA; (**D**) PolyT.

The transfer characteristics (I_D_–V_G_) of NCG-based devices (Figure 8) exhibit ambipolar behavior with a well-defined Dirac point, similar to VG devices, but with reduced modulation following functionalization. The sequential modification of the channel, involving PASE activation, ssDNA immobilization, and DNA hybridization, induces measurable but more moderate changes in both Dirac point position and channel current.

PASE functionalization leads to a slight shift of the Dirac point accompanied by a decrease in the minimum current, indicating mild p-type doping induced by π–π interactions with the graphene-derived surface. The immobilization of ssDNA introduces additional electrostatic effects, resulting in gradual variations in carrier density, although the magnitude of these changes remains limited compared to VG.

DNA hybridization produces a detectable electrical response, primarily reflected in the modulation of the minimum current rather than significant shifts in the Dirac point. This behavior suggests that, in NCG devices, the sensing mechanism is dominated by changes in charge distribution and dielectric environment at the interface, with a stronger capacitive contribution and reduced influence on the overall energy landscape of the channel.

Sequence-dependent effects are present but less pronounced than in VG. G–C systems exhibit slightly stronger modulation compared to A–T systems, consistent with higher duplex stability, but the overall amplitude of the response remains moderate. In particular, the electrical signal is mainly expressed through current variation rather than energetic displacement, indicating a more delocalized transport regime. These observations are consistent with the structural characteristics of NCG, which forms a compact and relatively continuous conductive network. As a result, charge transport is less sensitive to local interfacial perturbations that lead to improved stability and reproducibility, but have reduced sensitivity to biomolecular interactions.

These results highlight that the electrical response is strongly governed by the intrinsic transport regime of the channel material, with VG enabling amplified sensitivity and NCG providing enhanced stability. To further elucidate the origin of these differences, morphological characterization was performed in order to correlate the electrical behavior with the structural organization of the graphene-based interfaces.

The electrical parameters extracted at V_D_ = 2V (Table 5) reveal a systematically attenuated but reproducible response to sequential functionalization, consistent with the more continuous percolative transport regime of NCG (ξ ≈ 3.0 nm, lower N(E_F_), stable T_0_).

PASE functionalization produces variable modulation of I_ON_/I_OFF_ across devices increasing in NCG3 (53.0 → 158.7) and NCG5 (469.5 → 541.3), while decreasing in NCG10 (372.3 → 178.1) with minimal V_Dirac_ displacement in all cases. This behavior reflects the preferential adsorption of PASE at grain boundaries and nanocrystalline domain edges, which locally perturbs hopping pathways without inducing net electrostatic gating of the bulk conductive network. The absence of consistent Dirac point shifts confirms that, in NCG, PASE interaction is primarily capacitive rather than charge-transfer dominated.

ssDNA immobilization produces modest V_Dirac_ shifts (≤0.20 V) in most devices, with the notable exception of NCG3 (ΔV = −2.10 V upon PolyG immobilization), where the strong π–π stacking affinity of guanine for the graphitic surface results in dense, localized adsorption that directly perturbs the energy of hopping states. The primary electrical signature across all devices remains I_ON_/I_OFF_ modulation, indicating that ssDNA predominantly modifies the local dielectric environment of the NCG channel rather than introducing direct charge transfer consistent with the longer localization length of NCG, which reduces the sensitivity of individual hopping events to point-like surface charges.

DNA hybridization produces the most sequence-resolved response. The G–C system (NCG3) exhibits the largest overall Dirac point displacement (V_Dirac_ = −2.55 V, ΔV = −6.95 V from init), accompanied by a decrease in I_D_,min and an increase in I_ON_/I_OFF_ to 222.2, reflecting the high thermodynamic stability of the G–C duplex and its strong interfacial coupling with the NCG surface. Within the VRH framework, the rigid, charge-dense G–C layer modifies tunneling barriers between localized states, effectively increasing the local T_0_ and producing a measurable shift of the charge neutrality point. The C–G system (NCG10) shows the opposite trend: a monotonic decrease in I_ON_/I_OFF_ (372.3 → 78.9) with negligible V_Dirac_ displacement, indicative of capacitive dielectric screening as the dominant transduction mechanism. A–T systems (NCG8) display near-stable V_Dirac_ and I_ON_/I_OFF_ throughout all functionalization steps (77.7 → 94.9), reflecting the weak electronic coupling of A–T duplexes with the NCG network and the limited sensitivity of the longer hopping pathways to diffuse interfacial charge redistribution. The T–A system (NCG5) presents a distinct behavior: I_ON_/I_OFF_ remains stable through PASE (541.3) and ssDNA (473.0) stages, undergoing a marked reduction exclusively upon dsDNA formation (99.1). This indicates that duplex formation rather than individual functionalization steps triggers a reorganization of the molecular layer that increases dielectric screening of the back-gate coupling. Collectively, the NCG results confirm that, within the VRH–thermally activated framework, the longer ξ and lower N(E_F_) of NCG shift the dominant transduction mechanism from electrostatic gating toward capacitive dielectric modulation, relative to VG. The resulting electrical response is more stable and reproducible but less sensitive in absolute terms, with sequence discrimination achieved primarily through I_ON_/I_OFF_ modulation rather than V_Dirac_ displacement establishing NCG as a complementary platform to VG for label-free nucleic acid detection.

### 3.7. Morphological Characterization

**Figure 9:**
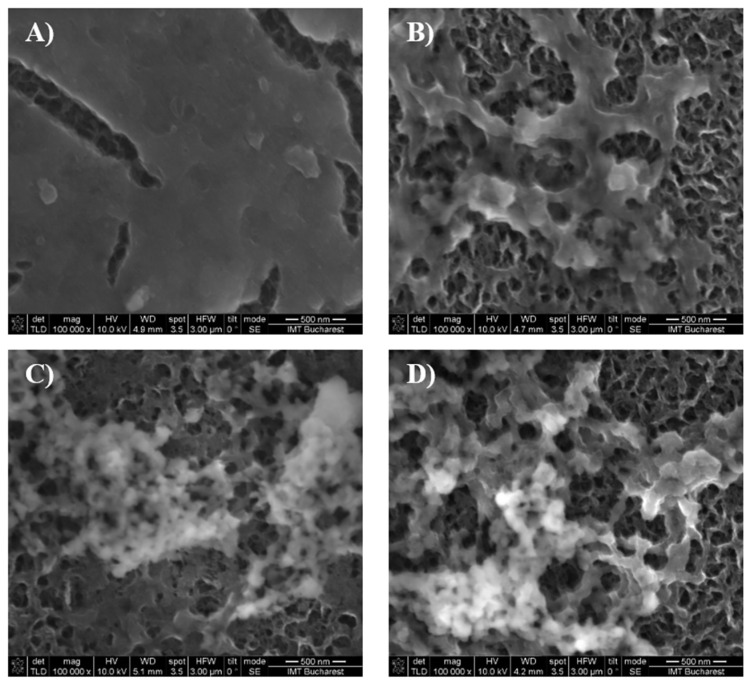
SEM images of VG functionalized with PASE and hybridized with DNA duplexes: (**A**) PolyG–PolyC, (**B**) PolyC–PolyG, (**C**) PolyT–PolyA, and (**D**) PolyA–PolyT, highlighting sequence-dependent molecular organization on the graphene surface.

In Figure 9, we present the SEM analysis of VG functionalized with PASE and hybridized with DNA duplexes. This analysis reveals the preservation of VG characteristic three-dimensional architecture, together with sequence-dependent interfacial organization. The structure, dominated by vertically aligned graphene sheets and a high density of exposed edges, remains clearly visible in all cases, confirming that functionalization induces controlled modification of the interface without disrupting the conductive network.

For G–C systems (Figure 9A,B), the surface exhibits localized compact domains and pronounced aggregation across the interconnected network. The high stability of G–C duplexes and stronger π–π interactions promote molecular accumulation at energetically favorable sites, particularly along edges and inter-sheet junctions. This localized densification is expected to enhance interfacial charge perturbation and modify the local potential landscape, consistent with the larger Dirac point shifts and stronger current modulation observed in electrical measurements.

In contrast, A–T systems (Figure 9C,D) display a more diffuse and homogeneous molecular coverage, with reduced aggregation and weaker local densification. This suggests a more uniform distribution of charge at the interface that results in a smaller perturbation of the electronic structure of the channel. Such behavior is in agreement with the reduced electrical response observed for A–T systems. Importantly, the open three-dimensional VG architecture remains accessible in all cases, which indicates that the conductive pathways are preserved while the interfacial region is progressively modified. This ensures efficient coupling between the molecular layer and the charge transport network.

**Figure 10:**
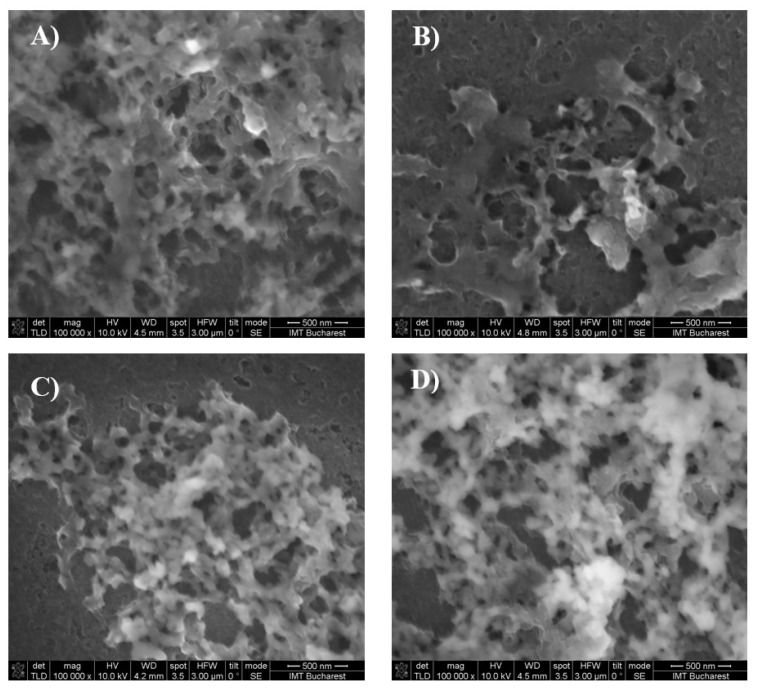
SEM images of NCG functionalized with PASE and hybridized with DNA duplexes: (**A**) PolyG–PolyC, (**B**) PolyC–PolyG, (**C**) PolyT–PolyA, and (**D**) PolyA–PolyT, highlighting sequence-dependent surface coverage on the compact graphene structure.

In Figure 10 we present the SEM analysis of NCG surfaces functionalized with PASE and hybridized with DNA duplexes. The analysis reveals a more compact morphology compared to VG, with a predominantly planar, granular structure. The surface is characterized by closely packed graphitic domains, providing a relatively continuous conductive network with limited vertical accessibility. For G–C systems (Figure 10A,B), localized molecular domains and moderate aggregation are observed, distributed over the granular matrix. Although G–C duplexes promote molecular accumulation, the extent of aggregation is less pronounced than in VG, suggesting that interactions are primarily confined to the surface. This results in a limited perturbation of the conductive pathways, consistent with the smaller Dirac point shifts observed in electrical measurements. In contrast, A–T systems (Figure 10C,D) exhibit a more uniform and continuous coverage, leading in some regions to partial surface densification. This behavior indicates the formation of a more compact molecular layer, which can influence charge transport mainly through dielectric effects rather than strong local charge perturbations. Such a uniform coverage is consistent with the observed electrical response, which is dominated by changes in current amplitude rather than significant energetic shifts.

Importantly, the overall morphology suggests that molecular functionalization occurs predominantly at the surface, without deep penetration into the conductive network. As a result, the interaction between the biomolecular layer and the charge transport pathways is weaker compared to VG.

These results demonstrate that the morphology of graphene-derived materials plays a critical role in defining the nanobiointerface and its electrical response. The enhanced interfacial coupling observed in VG, compared to the more surface-confined interaction in NCG, motivates further structural investigation in order to probe the underlying electronic modifications induced by functionalization. Therefore, Raman spectroscopy was employed to assess changes in the graphene lattice and charge distribution.

### 3.8. Structural Characterization by Raman Spectroscopy

Raman spectra recorded before and after functionalization confirm the preservation of the graphene-based structure, with the characteristic D (~1350 cm^−1^), G (~1580 cm^−1^), and 2D (~2700 cm^−1^) bands clearly defined. Following PASE activation and DNA immobilization, moderate variations in the I_D_/I_G_ ratio and slight shifts of the G band are observed, indicating changes in carrier density and molecular doping induced at the interface. The emergence or enhancement of a component around ~1620 cm^−1^ (D′ band), together with possible contributions associated with pyrene vibrations, further confirms the successful attachment of the molecular layer. Importantly, the absence of a significant increase in the D band intensity demonstrates that the functionalization process is predominantly non-covalent, preserving the integrity of the sp^2^ lattice.

**Figure 11:**
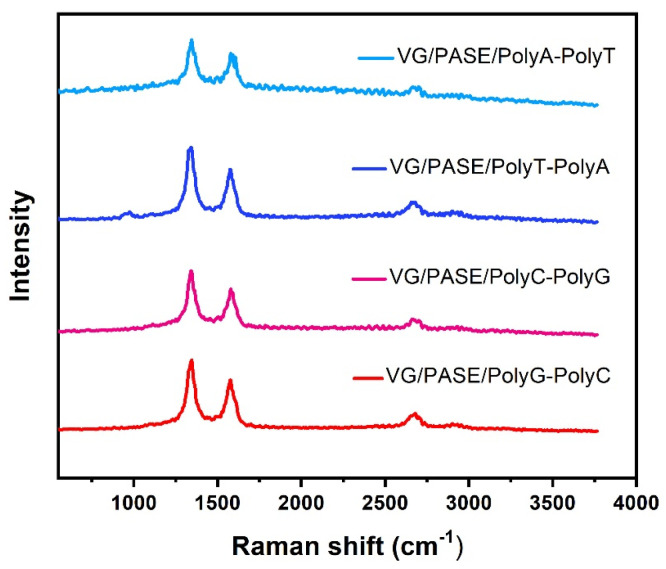
Raman spectra of VG/PASE/DNA duplex systems (PolyG–C, PolyC–G, PolyT–A, PolyA–T).

Raman spectra of VG/PASE/PolyX–PolyY systems is presented in Figure 11, and it exhibits a pronounced D band, a well-defined G band, and a broadened, less intense 2D band, characteristic of turbostratic multilayer graphene with a high density of exposed edges. The elevated I_D_/I_G_ ratio reflects the intrinsic structural disorder of VG, which facilitates molecular adsorption and enhances interfacial coupling. Despite functionalization, the absence of significant G band shifts confirms that the sp^2^ network remains structurally intact.

Sequence-dependent variations are more pronounced in VG, with PolyG–PolyC systems showing the strongest spectral modifications, indicative of enhanced electronic coupling and charge redistribution at the interface. In contrast, PolyA–PolyT systems exhibit weaker Raman changes, consistent with their reduced electrical response. These results demonstrate that, in VG, the Raman response is dominated by interfacial processes and edge-related effects, which play a key role in amplifying the electrical signal.

**Figure 12:**
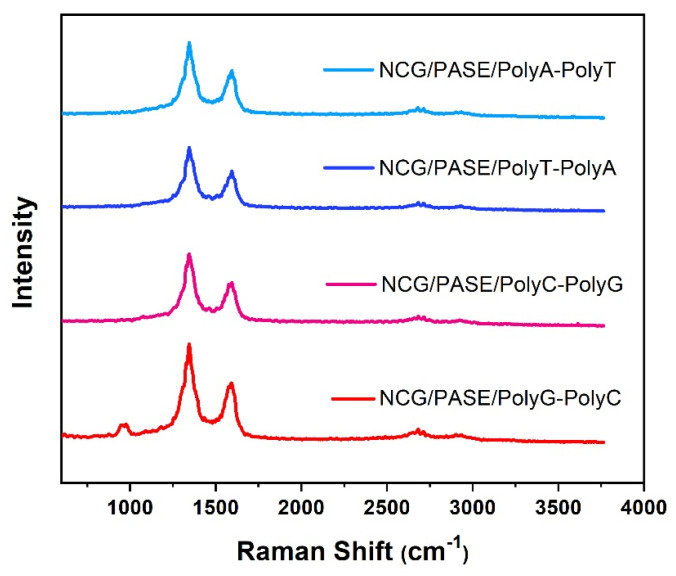
Raman spectra of NCG/PASE/DNA duplex systems (PolyG–C, PolyC–G, PolyT–A, PolyA–T).

In contrast, Raman spectra of NCG/PASE/PolyX–PolyY systems is presented in Figure 12, and it exhibits the characteristic features of nanocrystalline graphitic materials, with a relatively narrow and well-defined G band and a clearly resolved 2D band, indicating a higher degree of structural order compared to VG. The moderate I_D_/I_G_ ratio confirms the presence of nanocrystalline domains with controlled defect density, without structural degradation after functionalization and DNA hybridization. The stability of the G band position further supports the preservation of the electronic structure of the channel.

DNA duplex formation induces subtle but reproducible modifications in the Raman response of NCG, reflected by small variations in the D/G intensity ratio and minor shifts of the G band, associated with interfacial charge transfer and π–π interactions within the PASE–DNA–NCG system. These effects are more evident for PolyG–PolyC systems, suggesting a stronger electronic interaction of guanine with the graphitic surface compared to A–T duplexes. However, the overall magnitude of these spectral changes remains limited, indicating that biomolecular interactions are predominantly confined to the surface and induce only moderate perturbations of the electronic structure. This behavior is consistent with the electrical response of NCG, where smaller Dirac point shifts and current-dominated modulation were observed, reflecting a more stable but less sensitive transport regime.

These findings are in excellent agreement with both the morphological and electrical results. In VG, the three-dimensional architecture enables strong coupling between the molecular layer and the conductive network, leading to significant modulation of charge transport and pronounced Dirac point shifts. In contrast, the compact structure of NCG limits this interaction, resulting in a more stable but less responsive electrical behavior, where biomolecular effects are primarily reflected in current amplitude variations.

## 4. Conclusions

In the present work, we investigated the electron transport mechanisms of VG and NCG, two new state-of-the-art 3D nanocarbonic materials, as gate materials of a field effect transistor. To understand the underlying transport mechanism electrical characterization was performed by measuring current–voltage (I_D_–V_G_) transfer characteristics. Furthermore, we evaluated the interaction and influence of DNA onto charge transport mechanism of the two materials with the help of a DNA hybridization experiment.

Temperature-dependent electrical measurements (15–500 K) establish a hybrid transport regime in both VG and NCG: 3D Mott VRH below 240 K transitioning to thermally activated conduction above 240 K, placing both materials near the disorder-driven metal–insulator boundary.

Experiments show that several intrinsic transport parameters directly define the biosensing behavior of each platform. In VG, short ξ, high N(E_F_), and short R_hop_ amplify sensitivity to functionalization-induced modifications, producing pronounced sequence-resolved electrical responses in which G–C systems engage both electrostatic gating and transport modulation, while C–G and T–A systems operate through capacitive dielectric screening. In NCG, longer hopping distances shift the dominant mechanism toward semiconductor modulation, yielding attenuated but stable and reproducible responses. V_Dirac_ encodes net interfacial charge transfer while I_ON_/I_OFF_ encodes semiconductor modulation of the conductive network, with their relative contributions being sequence-specific and interpretable through ξ, N(E_F_), and T_0_.

This work establishes a quantitative, physically grounded correlation between disorder-driven transport and label-free nucleic acid detection, introducing transconductance and I_ON_/I_OFF_ as complementary experimentally accessible parameters linking VRH transport to BioFET sensitivity. The unified VRH–g_m_–sensing framework developed here provides a rational design principle for next-generation graphene BioFETs: engineering structural disorder, localization length, and edge density to optimize sensitivity, selectivity, and stability for targeted biosensing applications. The present study is limited to single-device measurements per condition; replicate statistics and non-complementary sequence controls are identified as necessary steps toward analytical validation in subsequent work.

## Figures and Tables

**Table 1:** Comparative overview of VRH conduction models and key transport parameters reported for representative disordered carbon-based systems.

Study	Material	VRH Model	Dim.	Dominant Regime	Key Observations
Sui et al. [22]	rGO paper (rGOP)	Mott VRH	2D	VRH at low T	2D planar structure → 2D VRH; dimensionality determined by assembly morphology
Sui et al. [22]	rGO aerogel fiber (rGAF)	Mott VRH	3D	VRH at low T	Artificial 3D nanostructure → 3D VRH; confirms morphology governs transport regime
Matsuda et al. [23]	Vertically aligned CNT films	VRH + graphitic conduction	3D	VRH (thin) → graphitic (thick)	CNT length governs VRH dimensionality; hybrid transport analogous to VG/NCG
Goto [24]	Graphitic carbon (PANI-derived)	Mott 3D VRH	3D	VRH (low T) + inter-domain hopping (high T)	Hybrid regime directly analogous to transport in VG and NCG channels
Gentoiu et al. [25]	Carbon nanowalls/NCG	Mott 3D VRH	3D	VRH dominant	Disorder–transport correlation; no biosensing application reported
Joung et al. [26]	RGO (sp^2^ = 55–80%)	ES-VRH	2D	ES-VRH (low T)	T_0_ and ξ strongly dependent on sp^2^ fraction; supports Mott 3D VRH for higher sp^2^ materials
Goh et al. [27]	SWCNT thin films	Mott VRH	3D	VRH dominant	SWCNT length < 700 nm → sharp T_0_ increase; *ξ* mirrors tube length
This work	VG	Mott 3D VRH + thermally activated	3D	VRH (T < 240 K) + Arrhenius (T > 240 K)	Disorder-dominated; enhanced current modulation upon DNA functionalization
This work	NCG	Mott 3D VRH + thermally activated	3D	VRH (T < 240 K) + Arrhenius (T > 240 K)	More continuous network; stable Dirac point shifts upon DNA functionalization

**Legend:** ES-VRH = Efros–Shklovskii VRH; Dim. = effective transport dimensionality.

**Table 2:** Oligonucleotide probe specifications (Integrated DNA Technologies, Inc. (IDT, Coralville, IA, USA)).

Probe	Sequence (5′→3′)	Length (Bases)	Modification
PolyG–NH_2_	5′-/5AmMC6/GGG GGG GGG GGG GGG GGG GG-3′	20	5′ Amino Modifier C6
PolyC–NH_2_	5′-/5AmMC6/CCC CCC CCC CCC CCC CCC CC-3′	20	5′ Amino Modifier C6
PolyA–NH_2_	5′-/5AmMC6/AAA AAA AAA AAA AAA AAA AA-3′	20	5′ Amino Modifier C6
PolyT–NH_2_	5′-/5AmMC6/TTT TTT TTT TTT TTT TTT TT-3′	20	5′ Amino Modifier C6

**Table 3:** Extracted VRH transport parameters for VG- and NCG-based FET structures.

Parameter	VG: ξ = 1.5 nm		NCG: ξ = 3.0 nm	
	T_max_ = 120 K	T_max_ = 240 K	T_max_ = 120 K	T_max_ = 240 K
T_0_ (K)	1697	3235	2021	2397
N(E_F_) (eV^−1^cm^−3^)	~4.3 × 10^22^	~2.2 × 10^22^	~4.5 × 10^21^	~3.8 × 10^21^
R_hop_ (nm)	2.91	2.87	6.08	5.33

**Table 4:** Electrical performance parameters of VG-FET devices upon sequential functionalization and DNA hybridization (VD = 2 V).

Device	System	State	V_Dirac_ (V)	ΔV_Dirac_ (V)	I_D_, min (µA)	I_ON_/I_OFF_
VG3	PolyG–PolyC	VG (init)	2.25	—	47.18	57.2
		PASE	2.35	+0.10	28.69	97.6
		ssDNA: PolyG	2.45	+0.20	55.29	47.0
		dsDNA: PolyG–C	2.35	+0.10	53.04	54.7
VG11	PolyC–PolyG	VG (init)	2.30	—	15.06	172.6
		PASE	2.30	0.00	12.83	210.5
		ssDNA: PolyC	2.30	0.00	31.29	89.5
		dsDNA: PolyC–G	2.30	0.00	61.37	48.9
VG8	PolyA–PolyT	VG (init)	2.30	—	59.68	26.8
		PASE	2.40	+0.10	44.50	33.7
		ssDNA: PolyA	2.50	+0.20	23.64	73.0
		dsDNA: PolyA–T	2.40	+0.10	19.18	71.9
VG6	PolyT–PolyA	VG (init)	2.35	—	6.09	263.2
		PASE	2.35	0.00	48.27	35.2
		ssDNA: PolyT	2.35	0.00	14.11	106.3
		dsDNA: PolyT–A	2.35	0.00	33.98	53.0

V_Dirac_ from minimum drain current in transfer characteristics; ΔV_Dirac_ relative to bare VG channel.

**Table 5:** Electrical performance parameters of NCG-FET devices after sequential functionalization and DNA hybridization (V_D_ = 2 V).

Device	System	State	V_Dirac_ (V)	ΔV_Dirac_ (V)	I_D_,min (µA)	I_ON_/I_OFF_
NCG3	PolyG–PolyC	NCG (init)	4.40	—	3.72	53.0
		PASE	5.00	+0.60	11.97	158.7
		ssDNA: PolyG	2.30	−2.10	22.20	94.6
		dsDNA: PolyG–C	−2.55	−6.95	8.10	222.2
NCG10	PolyC–PolyG	NCG (init)	2.50	—	3.76	372.3
		PASE	2.40	−0.10	8.14	178.1
		ssDNA: PolyC	2.45	−0.05	15.24	98.4
		dsDNA: PolyC–G	2.40	−0.10	20.28	78.9
NCG8	PolyA–PolyT	NCG (init)	2.45	—	28.30	77.7
		PASE	2.45	0.00	26.51	79.2
		ssDNA: PolyA	2.35	−0.10	21.64	92.4
		dsDNA: PolyA–T	2.30	−0.15	22.12	94.9
NCG5	PolyT–PolyA	NCG (init)	2.45	—	4.26	469.5
		PASE	2.30	−0.15	3.51	541.3
		ssDNA: PolyT	2.25	−0.20	4.44	473.0
		dsDNA: PolyT–A	2.30	−0.15	17.16	99.1

## Data Availability

The data presented in this study are available on request from the corresponding author. The data are not publicly available due to ongoing research activities.

## Abbreviations

The following abbreviations are used in this manuscript:

3D	Three-dimensional
2D	Two-dimensional
FET	Field-effect transistor
CNT	Carbon nanotube
CVD	Chemical vapor deposition
DMF	Dimethylformamide
DNA	Deoxyribonucleic acid
dsDNA	Double-stranded deoxyribonucleic acid
E_a_	Activation energy
EF	Fermi energy level
ES-VRH	Efros–Shklovskii variable range hopping
FEG-SEM	Field-emission gun scanning electron microscopy
HOMO	Highest occupied molecular orbital
I_D_	Drain current
I_D_,min	Minimum drain current
I_ON_/I_OFF_	On/off current ratio
ISO	International Organization for Standardization
IDTE	Integrated DNA Technologies elution buffer
k_B_	Boltzmann constant
MIS	Metal–insulator–semiconductor
N(E_F_)	Density of states at the Fermi level
NCG	Nanocrystalline graphite
NHS	N-hydroxysuccinimide
PASE	1-Pyrenebutyric acid N-hydroxysuccinimide ester
PANI	Polyaniline
PolyA	Poly-adenine (homopolymer DNA probe)
PolyC	Poly-cytosine (homopolymer DNA probe)
PolyG	Poly-guanine (homopolymer DNA probe)
PolyT	Poly-thymine (homopolymer DNA probe)
rGO	Reduced graphene oxide
rGAF	Reduced graphene oxide aerogel fiber
rGOP	Reduced graphene oxide paper
R_hop_	Mean hopping distance
SEM	Scanning electron microscopy
SiO_2_	Silicon dioxide
ssDNA	Single-stranded deoxyribonucleic acid
SWCNT	Single-walled carbon nanotube
T_0_	Characteristic temperature (VRH model)
T_m_	Melting temperature
V_D_	Drain voltage
V_Dirac_	Dirac point voltage
VG	Vertical graphene
V_G_	Gate voltage (context-dependent)
VRH	Variable range hopping
ξ	Carrier localization length
σ	Electrical conductivity
α	Temperature coefficient of resistivity
